# COVID-19 and Intractable Hiccups: An Apparently Harmless Yet Misleading Manifestation

**DOI:** 10.7759/cureus.65966

**Published:** 2024-08-01

**Authors:** Asma Albtoosh, Nizar S Alkhlaifat, Ahmad Aldurgham, Zeyad Fraij, Mohammed Aloqaily

**Affiliations:** 1 Respiratory and Internal Medicine, The University of Jordan, Amman, JOR; 2 Pediatrics, Western Michigan University Homer Stryker M.D. School of Medicine, Kalamazoo, USA; 3 Internal Medicine, King Hussein Medical City, Amman, JOR; 4 Medicine, The University of Jordan, Amman, JOR; 5 Internal Medicine, University of Maryland Midtown Campus, Baltimore, USA

**Keywords:** covid-19 and hiccups, persistent hiccups, coronavirus, covid -19, hiccups

## Abstract

COVID-19 infection typically presents with various symptoms encompassing fever, cough, and shortness of breath, with occasional reports of less common manifestations such as dizziness and persistent hiccups. Herein, we present a distinctive case wherein refractory hiccups constituted the exclusive complaint, persisting despite standard therapeutic interventions and serving as a misleading sole presentation for COVID-19 infection that, unfortunately, was complicated by septic shock and patient demise. This case prompts contemplation regarding the potential impact of hiccups on the prognosis of COVID-19. It also underscores the imperative nature of recognizing and considering atypical and uncommon presentations, as these may carry significant and even more severe consequences.

## Introduction

The coronavirus disease 2019 (COVID-19) was proclaimed a pandemic in 2020 by the World Health Organization (WHO) [1]. COVID-19, caused by SARS-CoV-2, spreads through respiratory droplets from coughing, sneezing, and speaking, primarily affecting the respiratory and vascular systems [1,2]. Most patients who contracted the virus presented with fever, cough, tiredness, and troubled breathing [2]. Nevertheless, COVID-19 patients could suffer and present with other uncommon and atypical manifestations such as chest pain, headache, dizziness, nausea and vomiting, diarrhea, and the newly discovered, refractory hiccups, albeit rarely [3,4].

The nature of hiccups, while usually harmless, is not fully understood, and some cases may indicate a serious underlying condition if hiccups are intractable [5]. The hiccup center involves areas of the central nervous system. Persistent hiccups lasting over two days, though usually benign, can signal underlying health issues such as central nervous system diseases, medical procedures, gastrointestinal disturbances, and even myocardial ischemia [6].

A recent systematic review that was published in 2022 has revealed only 16 reported cases of persistent hiccups linked to COVID-19, with most patients seeing improvement after treatment [7]. Treating persistent hiccups is challenging and may involve medications, interventional methods like phrenic nerve blocks, or in severe cases, general anesthesia or positive pressure ventilation with muscle relaxants. Often, a combination of treatments is necessary [8]. 

Herein, we present a case of an 82-year-old male who was diagnosed with COVID-19 infection after presenting with intractable hiccups, after which he suffered from catastrophic events.

This article was presented as pre-print on the following website: https://www.authorea.com/users/609239/articles/666646-covid-19-presenting-as-intractable-hiccups-an-apparently-harmless-yet-misleading-manifestation [9].

## Case presentation

An 82-year-old man with a medical history of hypertension, hyperlipidemia, chronic kidney disease (with baseline creatinine of 2.5 mg/dl, and Kidney Disease: Improving Global Outcomes (KDIGO) classification of G4/A1), Coronary Artery Bypass Graft (CABG), and Coronary Stent Placement presented at the emergency department with persistent hiccups for five days. He was a smoker with 50 pack-years and had allergies to metoclopramide and morphine.

The patient reported refractory hiccups lasting five days, possibly triggered by family members' upper respiratory infection. He also experienced a dry cough and orthopnea. There was no rhinorrhea, conjunctivitis, or abnormal breathing sounds. No other symptoms like fever, chest pain, or gastrointestinal issues were noted. His current medications include atenolol, enalapril, acetylsalicylic acid, and clopidogrel.

During the physical examination, the patient appeared comfortable with no significant findings except for bibasilar crackles. His vital signs included a fever (38.6°C) and borderline hypotension (97/55 mmHg), while oxygen saturation was 96% on room air. Subsequently, he received IV fluids and antipyretics.

**Figure 1:**
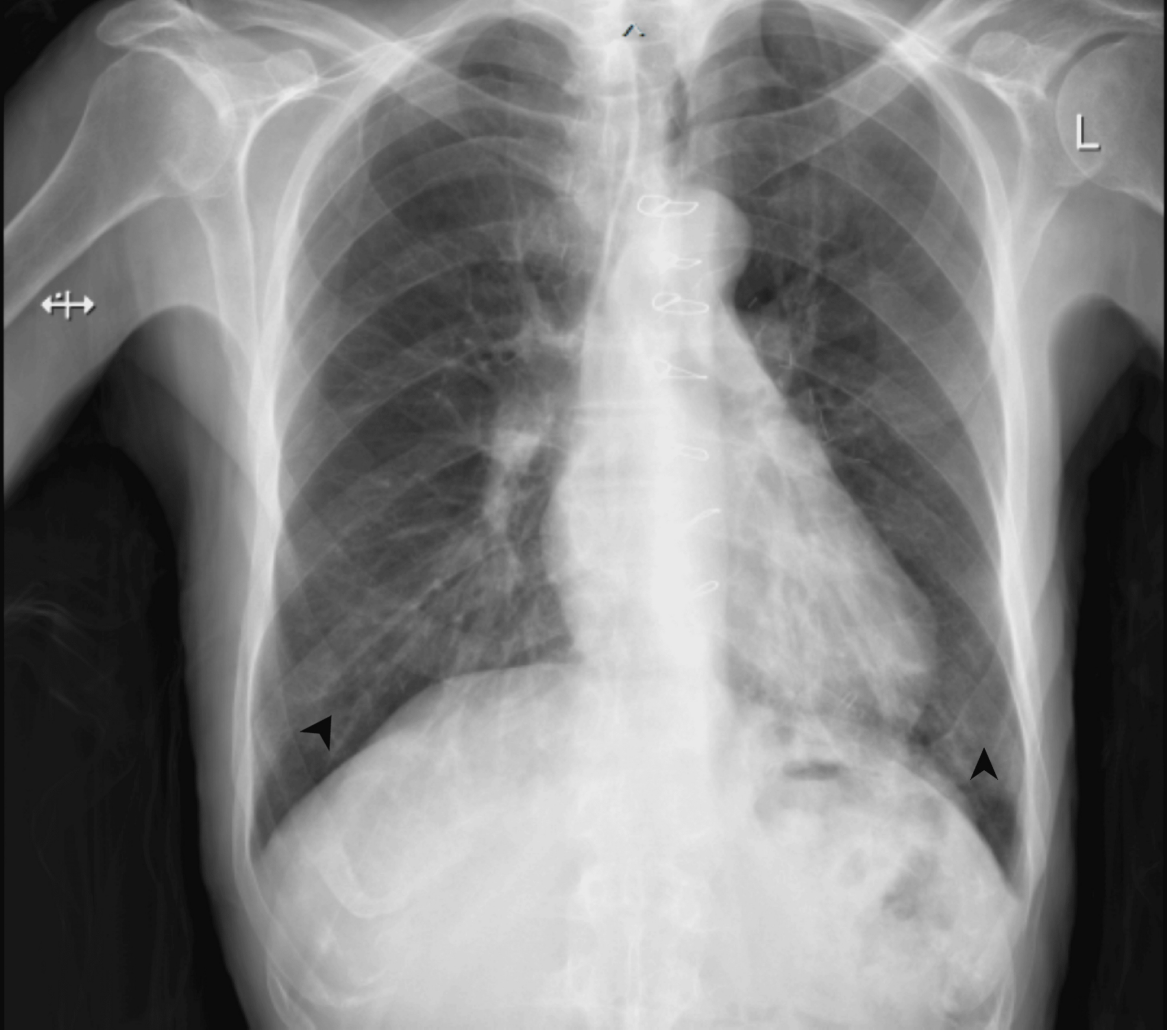
Chest X-ray upon presentation showing bilateral lower zones infiltrate indicated by the arrows.

The laboratory results indicated an elevated B-type natriuretic peptide (BNP) (544 pg/ml), WBC 7.78× 10^9^/L, neutrophils 89.1%, and lymphocytes 5%. Urine analysis showed no abnormalities (as demonstrated in Table 1). A chest X-ray revealed bilateral lower zone infiltrates (Figure 1), prompting blood and urine cultures. Despite fluid administration, the patient's blood pressure didn't improve, leading to initiation of noradrenaline infusion, IV antibiotics, and ICU admission.

**Table 1:** Important laboratory tests with some interval changes.

Laboratory test	Value
WBC (White blood cells)	7.78*10^9^ cell/L
Neutrophil %	89.1%
Lymphocyte %	5%
Hemoglobin	12 g/dl
BNP (B-type natriuretic peptide)	544 pg/ml > 752 pg/ml
Creatinine (baseline 2.5)	2.55 mg/dl > 1.5 mg/dl
C-reactive protein	230 mg/L (peak)
Urine analysis	Negative
LDH (Lactate dehydrogenase)	563 units/L
D-dimer	2.99 mg/L
Troponin	114
IL-6	192
COVID-19	Positive
Blood Cultures	Negative

Following admission, the patient experienced tachycardia (heart rate of 150 without ECG changes) and a decrease in oxygen saturation to 89%, necessitating oxygen therapy via a nasal cannula at 3L/minute. Additionally, his SARS-CoV-2 PCR test returned positive. Notably, the patient had not received a COVID-19 vaccination.

Despite extensive measures and COVID-19 management, the patient's blood pressure dropped again when a trial of weaning off noradrenaline was initiated after blood pressure was stabilized, it is worth mentioning that inotropes were not stopped during the whole period of hospitalization to maintain a mean arterial pressure of >65 mmHg. Furthermore, lab results worsened with low hemoglobin, elevated lactate dehydrogenase (LDH) (563 units/L), D-dimer (2.99 mg/L), C-reactive protein (peaked at 230 mg/L but decreased with IV antibiotics), IL-6 (192), and blood cultures were negative. Of notice, Creatinine improved to 1.5 mg/dl during his prolonged hospitalization course of over a month. It is worth mentioning that atrial fibrillation with rapid ventricular response emerged and was managed with rate-controlling medication and anticoagulation.

**Figure 2:**
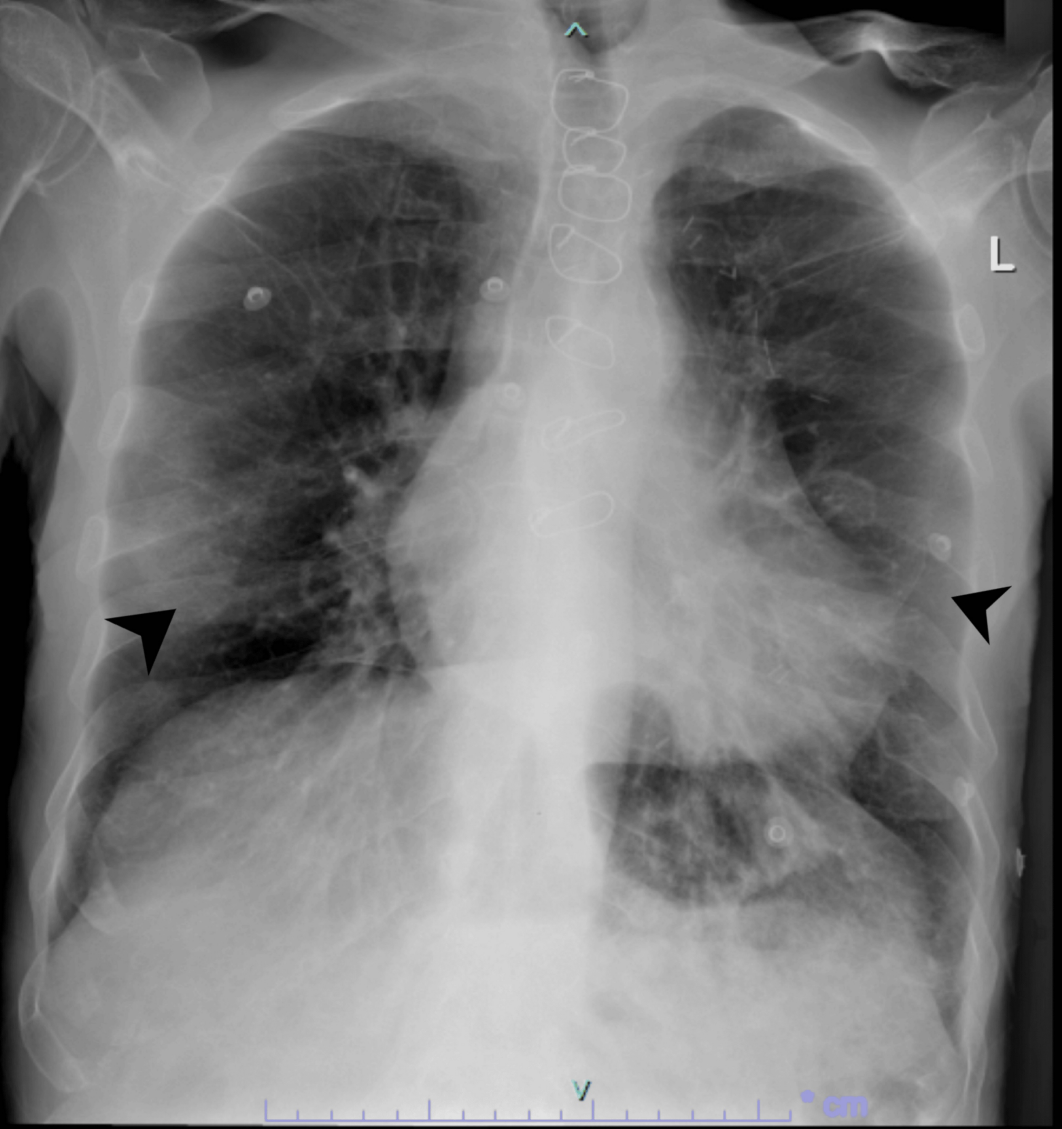
Chest X-ray after two days revealing more obvious bilateral peripheral infiltrates indicated by the arrows, characteristic of Covid-19 pneumonia.

**Figure 3:**
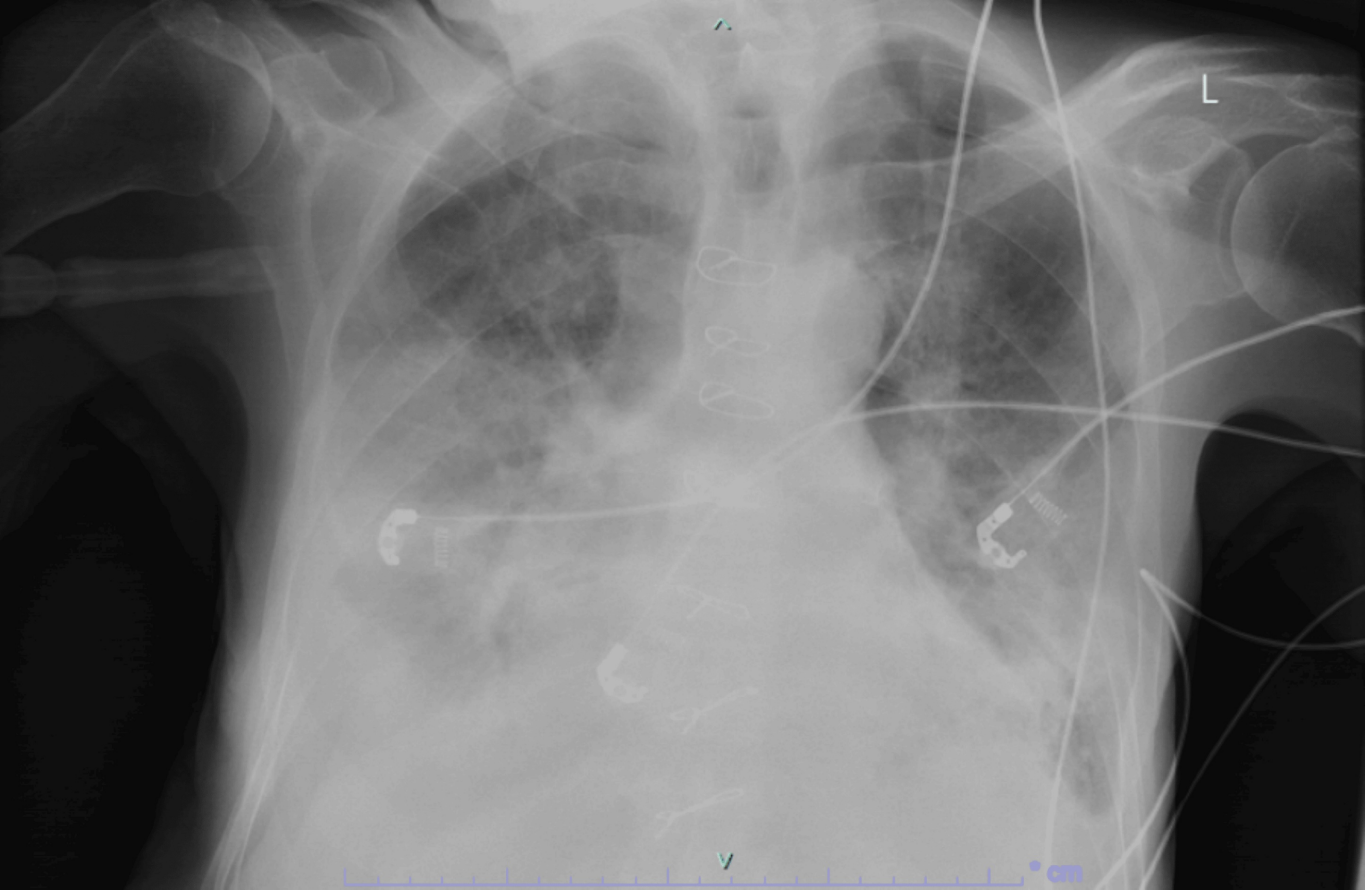
A few days later, chest X-ray deteriorated and continued to deteriorate after until he passed away, unfortunately.

Surprisingly, repeated chest X-rays showed worsening bilateral infiltrates and pulmonary vascular congestion over time (Figures 2, 3), and the patient began experiencing atypical chest pain. An ECG revealed new ST depression in leads V4-V6 and atrial fibrillation. Troponin levels were elevated at 114, and BNP was 752, leading to a diagnosis of Non-ST-Elevation Myocardial Infarction (NSTEMI). Treatment was initiated. Subsequent bedside echocardiography by a cardiologist indicated an ejection fraction of 45%, bilateral atrial enlargement, and minimal pericardial effusion.

Unfortunately, sudden cardiac arrest with asystole ensued while in the ICU, and CPR was started but the rhythm did not revert, and the patient was announced dead.

## Discussion

In March 2020, the WHO declared COVID-19 a global pandemic, causing significant global health, economic, and societal damage with millions of deaths [10]. COVID-19, caused by SARS-CoV-2, spreads through respiratory droplets, primarily from coughing, sneezing, and speaking, targeting the respiratory and vascular systems [11].

The hiccup center involves multiple areas of the central nervous system, including the phrenic nerve nuclei, the brainstem, the respiratory center, the hypothalamus, and the medullary reticular formation. Motor fibers of the phrenic nerve mediate the primary efferent pathway of this reflex [6]. Most hiccup spasms are unilateral, typically affecting the left hemidiaphragm [6].

Common COVID-19 symptoms include fever, cough, and breathing difficulties, but atypical symptoms like sneezing, hemoptysis, shivering, and persistent hiccups have been reported [12]. Although persistent hiccups lasting over two days are generally benign, they can indicate underlying health problems [13]. Such cases have been linked to central nervous system diseases or injuries (e.g., stroke, tumor, Parkinson's disease, multiple sclerosis), medical procedures and medications (e.g., anesthetic agents, chemotherapy, azithromycin, catheter ablation), gastrointestinal disturbances (e.g., gastroesophageal reflux disease, *Helicobacter pylori*, esophageal tumor), and occasionally myocardial ischemia [8]. The exact cause remains unclear but might involve nerve cell selectivity, irritation of nerves due to pneumonia, and arrhythmias [12-14].

Long-lasting hiccups in COVID-19 cases are rare, with only 16 reported cases in 2022, often overlooked in clinical contexts [5]. Some patients with persistent hiccups had elevated C-reactive protein, D-Dimer, LDH, and hypertension. For patients with refractory hiccups, comprehensive evaluations are needed to rule out other potential sources like gastroesophageal reflux disease, central nervous system disorders, and chemical imbalances [15]. CT scans and MRIs may be necessary in some cases [15].

Treating persistent hiccups can be challenging, but several approaches may be effective such as Pharmacologic options (metoclopramide, omeprazole, and baclofen), Interventional methods like phrenic nerve blocks, or in some cases, general anesthesia or positive pressure ventilation [16]. However, there is no universal solution, and a combination of strategies may be necessary. It is of high importance to mention that most cases of COVID-19-induced intractable hiccups were responsive to medical management using metoclopramide, chlorpromazine, and baclofen [5,7]. However, in our case, metoclopramide treatment was considered but was not possible due to the patient’s allergies and the subsequent deterioration leading to intubation.

Given the current COVID-19 pandemic, SARS-CoV-2 infection should be considered, even without common symptoms [17]. A timely diagnosis is crucial to curbing the virus's spread and starting appropriate treatment. This case emphasizes the need for vigilance for COVID-19, even if hiccups are the only symptom.

The unique aspect of this case is that the patient had refractory hiccups as the sole complaint, alongside borderline blood pressure and chest X-ray findings. Most patients with persistent hiccups improved with treatment [5,7], but this was the second case where the patient unfortunately passed away despite efforts and measures taken [18].

## Conclusions

For patients with intractable hiccups, a thorough examination is crucial to rule out underlying conditions like gastroesophageal reflux disease and neurological disorders. Amid the COVID-19 pandemic, clinicians should consider SARS-CoV-2 infection, even without typical symptoms, stressing the importance of swift diagnosis. This case underscores vigilance for COVID-19, especially when hiccups are the sole symptom. Notably, despite efforts, including treatment, this represents the second instance where the patient passed away, highlighting the challenges in managing such cases effectively.
